# Fetus Sound Stimulation: Cilia Memristor Effect of Signal Transduction

**DOI:** 10.1155/2014/273932

**Published:** 2014-02-26

**Authors:** Svetlana Jankovic-Raznatovic, Svetlana Dragojevic-Dikic, Snezana Rakic, Branka Nikolic, Snezana Plesinac, Lidija Tasic, Zivko Perisic, Mirjana Sovilj, Tatjana Adamovic, Djuro Koruga

**Affiliations:** ^1^Department of Obstetrics and Gynecology “Narodni front”, Kraljice Natalije Street 62, 11000 Belgrade, Serbia; ^2^Belgrade University Medical School, Doktora Subotica Street 8, 11000 Belgrade, Serbia; ^3^Institute for Obstetrics and Gynecology, Clinical Center of Serbia, Majke Jevrosime 8, 11000 Belgrade, Serbia; ^4^Institute for Experimental Phonetics and Speech Pathology, Gospodar Jovanova Street 35, 11000 Belgrade, Serbia; ^5^Life Activities Advancement Center, Gospodar Jovanova Street 35, 11000 Belgrade, Serbia; ^6^Biomedical engineering, Faculty of Mechanical Engineering, University of Belgrade, Kraljice Marije Street 8, 11000 Belgrade, Serbia

## Abstract

*Background*. This experimental study evaluates fetal middle cerebral artery (MCA) circulation after the defined prenatal acoustical stimulation (PAS) and the role of cilia in hearing and memory and could explain signal transduction and memory according to cilia optical-acoustical properties. *Methods*. PAS was performed twice on 119 no-risk term pregnancies. We analyzed fetal MCA circulation before, after first and second PAS. *Results*. Analysis of the Pulsatility index basic (PIB) and before PAS and Pulsatility index reactive after the first PAS (PIR 1) shows high statistical difference, representing high influence on the brain circulation. Analysis of PIB and Pulsatility index reactive after the second PAS (PIR 2) shows no statistical difference. Cilia as nanoscale structure possess magnetic flux linkage that depends on the amount of charge that has passed between two-terminal variable resistors of cilia. Microtubule resistance, as a function of the current through and voltage across the structure, leads to appearance of cilia memory with the “memristor” property. *Conclusion*. Acoustical and optical cilia properties play crucial role in hearing and memory processes. We suggest that fetuses are getting used to sound, developing a kind of memory patterns, considering acoustical and electromagnetically waves and involving cilia and microtubules and try to explain signal transduction.

## 1. Introduction

Prenatal acoustical stimulation (PAS) is a method of early detection of development of fetal auditory and behavioral function. It is based on a method of detecting fetal reactions on the defined sound stimulation [1]. However, the first idea of this method was proposed twenty years ago [2].

This experimental study evaluates fetal middle cerebral artery (MCA) circulation after the defined prenatal acoustical stimulation (PAS), the role of cilia in hearing and memory and explains signal transduction and memory according to optical and acoustical properties of cilia. Acoustical and optical (electromagnetic) properties of cilia play crucial role in this process. Under the influence of clustering water and interaction with microtubules, not only may cilia signal transduce from acoustical to electrical but also may have memory property.

Cilia are microtubule-based organelle, tiny hair-like structure that performs feats such as clearing microscopic debris from the lungs and determining the correct location of organs during development. Due to the importance of cilia functions for health, there is great interest in understanding the mechanism that controls the cilia beating patterns and signal transduction. Transduction of acoustical to electrical signal on molecular and organelles levels is challenging and is not well understood yet. Auditory and vestibular inner ear hair cells convert the mechanical stimuli (acoustical, gravity, and head movement) into electrical signals [3]. This mechanotransduction process is initiated by opening of mechanosensitive cationic channels near the tips of hair cell stereo cilia, but the identity of these ion channels is still unknown [4]. We assume that cilia, as one of the first natural biological devices with efficient signal transduction, play crucial role in signal transduction.

Mutations in over 30 different genes can lead to cilia defects and complex interactions among ciliopathy-associated proteins [5]. Genetic mutations compromising the proper cilia functioning are associated with defects in ciliogenesis and they form new class of diseases called ciliopathies [6].

They can affect many different organs and cause polycystic kidney disease [7], primary cilia dyskinesia, retinitis pigmentosa, polydactyly, brain malformations and hydrocephalus [8–11], blindness, anosmia [12], asthenospermia [13], obesity [14], cognitive deficits [15], and some syndromes: Bardet-Biedl syndrome [15–17], Kartagener's syndrome [16, 18, 19], and Senior-Løken syndrome [16]. There are also indications that the primary cilium is important in oncogenesis [15, 20], behavioral [21] and mental disorders [22]. Some findings imply that primary cilia play important roles in the earliest stages of embryonic development, which could be important in regenerative medicine [20].

The cilium is an organelle with chemosensory, photosensory, and mechanosensory function in various body tissues and it plays an important role in normal development. We wanted to give the new insight to its memory function, based on our current experiment and our previous mathematical model of converting acoustical to electrical signal [23] which results in changes of the cell behavior and physiology, according to primitive memory effect.

## 2. Materials and Methods 

Our study included 119 healthy pregnant women with no risk pregnancies, without complications of any kind, with the delivery at time. Our examination was performed in period from 37 to 41 weeks of gestational age. Gestational age was determined in relation to last menstruation and estimated by ultrasound examination. We examined 271 pregnant patients, but 52 of them did not meet inclusion criteria because the gestational week was beyond 37 gestational weeks. All women were informed about our procedures and they gave written informed consent before enrollment. Our examination has research Ethics Board Approval of Department of Obstetrics and Gynecology “Narodni front,” Belgrade, Serbia. Experimental study has been organized as a part of prospective experimental trial under the supervision of Ministry of Health and Education of Republic of Serbia, (2011–2014). Project included Belgrade University Medical School, Belgrade, Serbia (Department of Obstetrics and Gynecology “Narodni front” and Institute for Gynecology and Obstetrics, Clinical Center of Serbia), and Institute for Experimental Phonetics and Speech Pathology, Belgrade, Serbia. Modeling of microtubule and cilia biophysical properties has been done at the Department of Biomedical Engineering, Faculty of Mechanical Engineering, University of Belgrade, Serbia.

Fetal examination starts with standard ultrasound examination. Noise-canceling headphones types EP-107 are put on women's head, to cancel the influence of mother's acoustical stimulation. Fetal head and ear position near the mother's abdominal wall are determinate and the speaker is positioned 5 cm from abdominal wall, to the direction of fetal ear. The circle of Willis is easy to identify with B-scan and blood flow using color Doppler. Using data of blood flow through fetal middle cerebral artery (MCA), Pulsatility index before sound stimulation (PIB) can be measured [24].

The fetus is exposed to the digitalized generated sound stimulus performed by loudspeaker sets 5 cm away from abdominal wall. This sound is 90 dB of intensity, frequency range is 1500–4500 Hz, and the duration is 0,2 s. This sound stimulus is presenting once in order to investigate changes in cerebral circulation of the fetus. Color Doppler ultrasound is used to identify middle cerebral artery flow after sound stimulation and identify the PI index after it. We also measure the time in seconds from the stimulus to the measured values of Pulsatility index after stimulation (PIR) and identify reactivity. Doppler analysis of blood flow is performed on the Toshiba Nemio with the possibility of Doppler and color Doppler and convex sector probe with the frequency of 3.5 MHz. We examined blood flow, which is registered in the first third of middle cerebral artery from branching. For the analysis of the wave, we used Pulsatility index (PI):

(1) $$\begin{array}{c} \text{PI}=\frac{\left(S-D\right)}{M}, \end{array}$$

one of the major Doppler-parameter [25]. Pulsatility index (PI) is equal to systolic (*S*) minus diastolic (*D*) amplitude value of arterial waveform, divided by the mean (*M*) value of the area under the waveform. This parameter is considered as an indicator of the size of the peripheral resistance and belongs to one of the Doppler-indexes of peripheral vascular resistance. Measured values of PI before (PIB) and few seconds after exposure to define digitalized generated sound stimulation (PIR 1) indicate changes in the fetal cerebral circulation. If the PI values after acoustical stimulation (PIR) are lower compared to the basic values of this index before stimulation (PIB), there is an increase in blood flow of fetal middle cerebral, while in the case of higher values of PIR compared to basic ones (PIB) signify reduction in blood flow in examined middle cerebral artery [1].

Five minutes after first stimulation, we exposed fetus to the same sound stimulus and measured the Pulsatility index (PIR 2) of the fetal MCA.

We tested the changes of PI values, basic one (PIB), after first (PIR 1) and after second stimulation (PIR 2), because we assume that fetuses develop own memory functions and did not react on the repeated stimulus in the same way. We believe that cilia and microtubules as acoustical and electrical organelles are responsible for signal transduction with memory and learning properties [26].

Statistical analysis of two-way comparisons was done using 2-tailed Student's *t*-tests. *P* values of less than 0.05 were considered significant.

## 3. Results 

Study included 119 healthy pregnant women with no risk pregnancies, without complications of any kind, with the delivery at time. Our examination was performed in period from 37 to 41 weeks of gestational age.

The mean gestational age in weeks is 39.02. Age of pregnant women showed that the mean age is 30.2 years and the median is 30. The eldest one was 44 years old and the youngest was 18 years old. Only 0.8% of patients were younger than 20 years and 1.7% elder than 40. The majority of patients (69.7%) is at the age between 25 and 34 and could represent our population.

The analysis of the Pulsatility index basic (PIB) of fetal middle cerebral artery (MCA), before PAS, has shown that the mean PIB value is 1.4747 and median is 1.47. Maximal PIB value is 2.59, and minimal one is 0.78.

Pulsatility index reactive after the first sound stimulation (PIR 1) of fetal MCA has shown that the mean PIR 1 value is 1.3644 and median is 1.33. Maximal PIR 1 value is 2.57, and minimal one is 0.76.

Pulsatility index reactive after the second sound stimulation after five minutes (PIR 2) of fetal MCA has shown that the mean PIR 2 value is 1.4605 and median is 1.46. Maximal PIR 2 value is 2.47, and minimal one is 0.8.

Comparison of the Pulsatility index basic (PIB) and Pulsatility index reactive after the first sound stimulation (PIR 1), using *t*-test (*t* = 4.445, *P* < 0.01), showed that there is high statistical difference. It means that there is a high influence of first sound stimulation on the fetal brain circulation. Values of PIB and PIR 1 of fetal MCA are graphically presented at Figure 2.

Statistical analysis of the Pulsatility index basic (PIB) before the sound stimulation and Pulsatility index reactive after the second sound stimulation (PIR 2) of fetal MCA, according to *t*-test (*t* = 1.506, *P* > 0.05), has shown that there is no statistical difference (Figure 3).

We suggest that our fetuses are getting used to the sound stimulus, developing a kind of memory patterns, which are connected acoustical and electromagnetic waves involving cilia and microtubules.

Statistical analysis of the Pulsatility index reactive after the first sound stimulation (PIR 1) and Pulsatility index reactive after the second sound stimulation (PIR 1) of fetal MCA, using *t*-test (*t* = −5.347, *P* < 0.01), has shown that there is high statistical difference. It means that reaction after second sound stimulation is different, because fetuses from no risk pregnancies get used to it and could develop memory patterns.

## 4. Discussion

Cilia are microtubule-based organelles surrounded by membranes that protrude from the cells [6]. Inside cilia is a microtubule-based cytoskeleton which acts as scaffolding for various protein complexes and provides binding sites for molecular motor proteins such as kinesin II that help carry proteins up and down the microtubules [27].

Cilia are classified into three categories: motile, primary, and nodal [16].

1. Motile cilia are situated on the surface of nearly every cell of mammalian body and they are involved in fluid and cell movement (mucociliary clearance in the lung, cerebrospinal fluid movement in the brain, and ovum and sperm transport along the reproductive tracts). Motile cilia consist of 9 doublet microtubules surrounding 2 inner singlet microtubules (9 + 2), used to generate forces to induce motility [28].
2. Primary cilia are solitary organelles projecting from the surface of cells and they lack the central pair of microtubules needed to generate motile force, so they are described as having a 9 + 0 pattern. Primary cilia on epithelial cells provide chemosensation, thermosensation, and mechanosensation of the extracellular environment by playing “a sensory role mediating specific signaling cues, including soluble factors in the external cell environment, a secretory role in which a soluble protein is released to have an effect downstream of the fluid flow, and mediation of fluid flow if the cilia are motile” [29].
3. Nodal cilia are located at the node in gastrulation-stage embryos. They are solitary organelles that contain 9 + 0 microtubule architecture and possess the ability to move in a propeller-like way. Mutations that disrupt nodal cilia in mice indicate that they play essential roles in establishing signaling events required for specification of the left-right body axis in mammals [30].

Bearing in mind the complexity of cilia, most previous studies of cilia were attempting to study the signal transduction. Nine pairs of microtubules are arranged along the periphery, and one pair of microtubules is situated at the center. Microtubules are biopolymer filaments, hollow rods approximately 25 nm in outer diameter and 14 nm inner diameters. The tubular structure is made up of two polypeptides: alpha-tubulin and beta-tubulin [31]. Tubulin subunits (*α*, *β*) oscillate in microtubule protofilaments generating optical (electrical and magnetic) and acoustical oscillations [32]. Since mass of *α* and *β* subunits have small difference, only four amino acids, optical and acoustical oscillatory modes are coupling [23]. The nine outer pairs of microtubules are connected by nexin links. Also, each of the outer pairs is linked to the central pair by a radial spoke. Closer inspection of the outer microtubules shows that they have side arms of proteins, called dyneins. These proteins are actually mechanical motors and they are instrumental in cilia movement. The structure of cilia is much like a tube, and its long fibers are composed of microtubules. These microtubules often pair up to form doublets, which mathematical sign eight, since the two microtubules stick together along a line. Nine doublets form the larger ring that is known as the 9 + 2 pattern. When kinesin binds to one side of the doublets and not the other, the cilium flexes and curves, similar to the way our skeletal muscles contract. “In effect, cilium is a nanomachine composed of perhaps over 600 proteins in molecular complexes, many of which also function independently as nanomachines” [33].

Hair cells are the sensory receptors of the organ of Corti that is located in the scala media. Two types of hair cells are present in the human cochlea: inner hair cell and the outer hair cells. Only outer hair cells are in direct contact with the tectorial membrane [34]. Ciliated columnar epithelial cells have 200 to 300 hair-like protrusions called cilia. Cells are interconnected via desmosomes and tight junctions, creating a semipermeable membrane that is more selective than membrane found in other types of cell [34]. Auditory and vestibular inner ear hair cells convert the mechanical stimuli (acoustical, gravity, and head movement) into electrical signals. This mechanotransduction process is initiated by opening of mechanosensitive cationic channels near the tips of hair cell stereo cilia, but the identity of these ion channels is still unknown [4].

**Figure 1:**
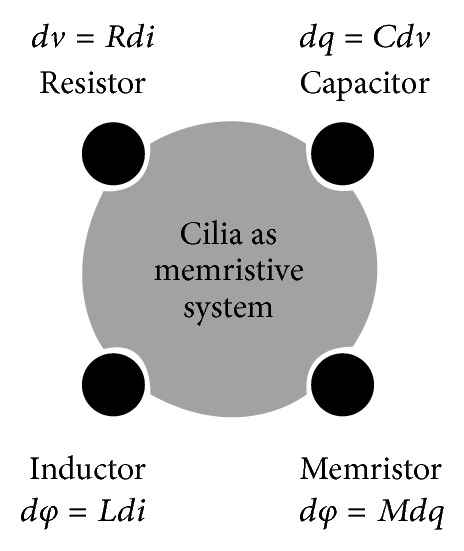
RCLM (resistance, capacitance, inductance, memory) circuit system plays crucial role in cilia dynamics (microtubules, water interaction). *dv*: *Rdi*: resistance, *dq*: *C* *dv*: capacitance, *dφ*: *Ldi*: inductance, and *dφ*: *M* *dq*: memory.

**Figure 2:**
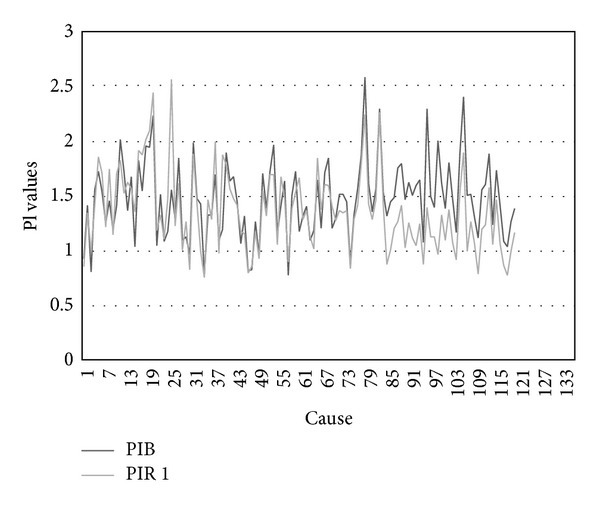
PIB and PIR 1 of fetal MCA values. PIB: Pulsatile index basic; PIR 1: Pulsatile index reactive after first sound stimulation.

**Figure 3:**
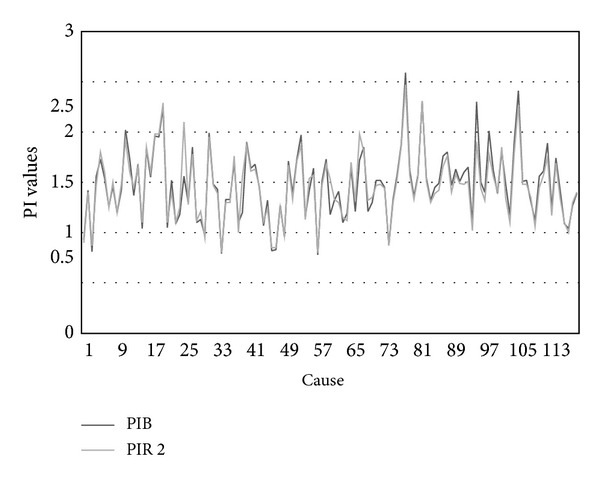
PIB and PIR 2 of fetal MCA values. PIB: Pulsatile index basic; PIR 2: Pulsatile index reactive after second sound stimulation.

New experimental system should be comprised of three main components: microtubule filaments, motor proteins called kinesin, which consume chemical fuel to move along microtubules, and a bundling agent that induces assembly of filaments into bundles. Based on knowledge about acoustical-electrical compatibility of cilia it is possible start to create artificial cilia-like structures that dramatically offers a new approach for cilia study and new sensory system for sensing reactions of fetus on the defined sound stimulation. The main approach to build new sensory system is to include memory effect in sensing system because cilia in process of open-close channel have “memristor” effect. By using only these three elements, resistor—*R*, capacitor—*C*, and inductor—*L*, this phenomenon could not be explained. The fourth element memristor *M* has to be included (Figure 1).

For cilia open-close channel process memristors, applied current, or voltage will cause a great change in resistance. Cilium may be characterized as switches (on-off) by investigating the time and energy that must be spent in order to achieve a desired change in resistance. Here we will assume that the applied voltage remains constant and solve for the energy dissipation during a single switching event. For a memristor to switch from *R*_*on*⁡_ to *R*_*off*⁡_ in time *T*_*on*⁡_ to *T*_*off*⁡_, the charge must change by Δ*Q* = *Q*_*on*⁡_ − *Q*_*off*⁡_:

(2) Eswitch=V2∫Toff⁡Ton⁡dtM(q(t))=V2∫Qoff⁡Qon⁡dqI(q)M(q)=V2∫Qoff⁡Qon⁡dqV(q)=VΔQ.

To arrive at the final expression, substitute *V* = *I*(*q*)*M*(*q*) and then ∫*dq*/*V* = Δ*Q*/*V* for constant *V*. This power characteristic differs fundamentally from that of a microtubule-water charges which are a capacitor-based device. Unlike the transistor, the final state of the memristor in terms of charge does not depend on bias voltage.

The idea of “memristor” existence, as a fourth-elementary circuit element (resistor, capacitor, and inductor) came from Leon Chua in 1971, strongly supported in 2004 [35], and finally experimentally proved in 2008 [36]. Using water, it is shown that memristance arises naturally in clustering water (nanoscale systems) in which cluster “solid state electronic” and ionic transport in microtubules are coupled under cilia oscillatory process. According to optical/acoustical values of cilia dynamics clustered water interacts with electrical charges on microtubules surface making RCLM (resistance, capacitance, inductance, memory) circuit system.

Fetus reactions on the defined sound stimulation in PAS can be better understood if we include cilia and microtubules as acoustical-electrical organelles. Under influence of clustering water and interaction with microtubules, not only cilia may signal transduce from acoustical to electrical, but also may have memory property. This process is self-induced by cilia. Paramecium and other one-cell organisms, which are very rich with cilia, possess both adaptive behavior and first step of learning. To explain signal transduction and memory based on cilia properties, optical/acoustical dynamics of microtubules during cilia motions is presented. Vibration spectra of microtubule indicate that cilia, as nanoscale structure (microtubules in interaction with water) possess magnetic flux linkage (Φ_*m*_), which depends on the amount of charge *q* that has passed between two-terminal variable resistors of cilia (nine pairs of microtubules). In this case microtubule resistance is a function of the history of the current through and voltage across the structure. This leads to appearance of cilia memory (*M*) based on microtubule resistance with the “memristor,” *M* = *d*Φ_*m*_/*dQ* property. Taking this knowledge about cilia properties, we are proposing new possible explanation of acoustical to electrical signal transduction under the defined sound influence on fetus during PAS. Statistical similarity of PIB and PIR 2 values, which represents no statistical changes (*t* = 1.506, *P* > 0.05) in fetal cerebral circulation after second defined acoustical stimulation is presented. This indicates that fetus auditory system possesses memory property and auditory screening is memorized.

## 5. Conclusions

Acoustical and optical (electromagnetic) properties of cilia play crucial rule in hearing and memory process.

Better understanding of information processing on biomolecular (microtubules), organelle (cilia), cell (hair cells) levels, and cell-nerve relationship within the organ of Corti could give us more information about fetal hearing, behavior, adaptation and memory (differences between PIB and PIR 1 and similarity between PIB and PIR 2), learning, and left-right side development.

We propose a new possible explanation of acoustical to electrical signal transduction under the defined sound influence on fetus during PAS, according to memory property of fetus auditory system.

The PAS method could be important for early detection of fetal auditory function during its development and help to establish reliable hearing screening test.
